# Conservative Surgery

**Published:** 1878-05

**Authors:** John E. Walker

**Affiliations:** Greenesboro, Ga.


### CONSERVATIVE SURGERY.

By JOHN E. WALKER, M.D., Greenesboro, Ga.

Case 1.—John B. Casselan, æt. 40, while tending a cotton gin, run by steam, had his sleeve caught, and his right hand and arm drawn in contact with the saws. The metacarpal bone of the thumb was exposed, that of the index finger was entirely divided, obliquely, and the metacarpo-phalangeal joint of the middle finger laid open; the fore-arm was lacerated in a dreadful manner, and, near the axilla, the skin and cellular tissue were torn off the arm, making a ragged wound, three by five inches, exposing the muscles. The index finger would have been removed but for the wound last described, which it was thought might involve the loss of the limb at the shoulder joint. The hand was adjusted with sutures, plaster and splints, as well as the circumstances would admit. The treatment consisted of solution of carbolic acid, which was constantly applied, by means of soft cloths—the fingers were semiflexed, and a ball of cotton placed in the palm for support—an anodyne at night to procure rest. An excellent cure resulted. The hand performed every function necessary, the index can be extended by the aid of the middle finger. The subject commands first-class wages as a laborer, or railroad track repairer.

Hamilton Jackson, colored, æt. 18, was caught by a loose car-brake, while attempting to couple a train, and received injuries of the left arm as follows, viz: compound fracture of humerus near elbow, a laceration just over the elbow joint, and along the front of forearm and hand, compound fracture of middle finger at the second joint. The flesh was torn up in the palm, exposing the fractured bone. I have been informed that an amputation was decided on, but the father, who had been summoned by telegraph, refused to allow it, and brought him to his home in Greenesboro, where he was put in my charge. I placed the arm and hand in a gutta-percha splint, curved at an obtuse angle, and kept the whole limb constantly wet with solution of carbolic acid. In five weeks the patient was up, and, in seven, hired as a laborer at half wages—he now commands the pay of a first-class hand. There is partial anchylosis of fingers, but they are so flexed that he can grasp any tool or implement; the motion of elbow not perfect, but sufficient for all practical purposes. The mistake of keeping fingers in a straight splint for like injuries, often occurs, which renders them not only useless, but an incumbrance.

Sam. Colb, colored, æt. 70, has had for a long time past an irreducible scrotal hernia. When seen, had been suffering with retention of urine for two days. Catheterism attempted, which failed, after persevering more than an hour. The bladder was greatly distended and the pain excruciating. Believing the difficulty was caused by an enlarged prostate, I decided that relief could only be obtained by puncture, but this was objected to by patient, and I left him unrelieved. In two days I was again called, and found the patient willing to submit to anything that promised relief. I again tried the catheter, with no better success. I then tried a male steel sound, which, after some delay, passed into the bladder; it was allowed to remain for a short time, then removed and the catheter again tried, which was introduced with very little difficulty, and the bladder relieved. This was repeated in the afternoon, and then twice daily for forty days, at the end of which time there was stilicidium, which increased until the use of the catheter was no longer necessary. In a short time he was able to retain and pass the urine at will. This old man lived more than two years, and died from other disease—never having had a return of the urinary difficulty. I have since used the same practice in other cases with equal satisfaction, sometimes employing the metalic bougie instead of the steel sound.

